# Caregiver Concerns for Children and Adolescents With Down Syndrome: A Cross‐Sectional Study in Brazil

**DOI:** 10.1111/cch.70283

**Published:** 2026-05-01

**Authors:** Beatriz Helena Brugnaro, Henrique Granado Jábali, Rosa Isabel Fonseca Angulo, Rafaela Campos, Marilyn Wright, Rachel Teplicky, Peter Rosenbaum, Olaf Kraus de Camargo, Nelci Adriana Cicuto Ferreira Rocha

**Affiliations:** ^1^ Department of Physical Therapy, Child Development Analysis Laboratory (LADI) Federal University of São Carlos (UFSCar) São Carlos São Paulo Brazil; ^2^ CanChild Centre for Childhood‐Onset Disability Research McMaster University Hamilton Canada; ^3^ Department of Pediatrics McMaster University Hamilton Ontario Canada

**Keywords:** About My Child, concerns, Down syndrome, families, functional concerns, participation

## Abstract

**Aim:**

The aim of this study is to describe and compare areas of concern for caregivers of children and adolescents with Down syndrome across ages and explore how these areas impact their ability to participate in daily activities.

**Methods:**

One hundred and seventeen caregivers of children and adolescents with Down syndrome, aged 0–18, participated and completed the About My Child questionnaire. Descriptive analyses of data from the caregivers were carried out. Individual item scores and the mean scores, standard deviations, median and confidence interval of the total Concern and Impact scores were calculated. Item analyses were carried out across age groups (Kruskal–Wallis test) and between children and adolescent groups (Mann–Whitney Test), aiming to explore concerns and impacts at different ages.

**Results:**

The lowest mean concern score for an age group was 9.27, and the highest was 12.67, with possible scores from 0 to 19. The lowest mean impact score for an age group was 2.80, and the highest was 3.25, with possible scores from 0 to 4. The items with the highest frequency of concern for most age groups were communication, participation in school and community and behaviour. The biggest impacts on participation were reported for the items concerning the use of arms and hands, sleep and hearing. No differences were found across age groups.

**Interpretation:**

This study found that caregivers of children and adolescents with Down syndrome have concerns about their child that, in total, do not change throughout life.

## Summary

- Caregivers are more concerned about communication, participation in school/community and behaviour.
- The biggest participation's impacts were on the use of arms/hands, sleep and hearing.
- There are no differences between the concerns of parents of children and parents of adolescents with Down syndrome.

## 1 Introduction

Down syndrome (DS), also known as trisomy 21, is a genetic condition that arises from the failure of chromosome 21 separation during gametogenesis. The estimated prevalence of this condition in Brazil is 4 per 10 000 live births (Laignier et al. 2021). Individuals with DS experience various impairments in body structures and functions, such as hypotonia, muscle weakness, ligament laxity, decreased functional mobility, impairments in motor coordination, postural control, heart disease and issues with cognitive and sensory processing (Santoro et al. 2020; Brugnaro et al. 2024; King et al. 2013).

In addition to these impairments, individuals with DS also experience social challenges (Imms 2021) and difficulties in their daily routines. Therefore, the primary caregivers of individuals with DS may be concerned about their ability to live independently (AlShatti et al. 2021). The quality of life of children with DS is impacted by their healthcare needs and can also impact the well‐being of the families (Hendrix et al. 2021; Williams et al. 2018) and the primary caregiver (AlShatti et al. 2021). According to some studies (Brugnaro et al. 2024; AlShatti et al. 2021), primary caregivers may initially face challenges in accepting the diagnosis, which leads them to seek answers to many questions about raising a person with DS. For children with developmental conditions, daily routines can be challenging due to recurring hospital stays and the necessity for specialized care, including assistance with mobility and maintaining sleep patterns, which also impacts the primary caregivers (Freitas et al. 2017).

In recent years, several structured support systems have been developed to promote the health, well‐being and participation of children and adolescents with DS and their families. These systems include early intervention services, family‐centred care models and community‐based initiatives. They aim to provide caregivers with social, educational and emotional support (Skotko et al. 2011; Van Riper et al. 2018).

Recent evidence emphasizes the importance of parent‐mediated interventions and culturally embedded support strategies as key factors for achieving positive outcomes in participation as well as functioning of children and adolescents with DS. In one study (Van Riper et al. 2018), it was reported that family resources and appraisal are strong predictors of adaptation, while (Stojanovik et al. 2024) another study demonstrated the feasibility of early social‐communication programmes (ASCEND) for young children with DS. Other studies highlight family self‐support (Noroozi et al. 2024) and caregiver experiences that reinforce the need for accessible resources and integrated systems of care (Zhang et al. 2025).

Complementary evidence from systematic reviews on family‐centred early intervention and caregiver quality of life further supports the role of coordinated services (McCarthy and Guerin 2022; Cheok et al. 2024; Rutter et al. 2024). In the Brazilian context, empirical studies have documented how social networks and family resources influence parental well‐being and adaptation among families of children with DS (Oliveira et al. 2024; Montes et al. 2023), including challenges related to social stigma that children and adolescents with DS face in their daily lives (Amorim and Shimizu 2022).

Additionally, earlier national research on family functioning and perceived support networks also provides essential background for understanding system‐level supports in Brazil (Rooke et al. 2019; Ronca et al. 2019).

Even though the literature regarding support systems highlights both international and Brazilian experiences, few studies have explored how caregivers' concerns evolve as children and adolescents with DS grow and develop.

Studies of children with physical disabilities report that parents' expressed concerns are higher among families and primary caregivers with lower incomes, and that the high proportion of parents with concerns about their children's development is alarming (Pratte et al. 2020). Regarding participation, caregivers face different difficulties in accessing medical care and rehabilitation therapies and negotiating with school and vocational services; these challenges are compounded with economic problems (Gokhale 2021). Concerns can evolve according to the individual's developmental stage (Cabrera et al. 2022). Most studies that analyse caregivers' concerns are in individuals with physical disabilities (Freitas et al. 2017; Pratte et al. 2020; Shuleski et al. 2022) rather than those with DS (AlShatti et al. 2021; Cabrera et al. 2022). In addition, most studies were qualitative in design, making it difficult to generalize the outcome to children and adolescents with DS (Gokhale 2021; Cabrera et al. 2022; Mkabile et al. 2021). Understanding the perceptions and concerns of families is an important factor in care provision, which can impact the quality of life of the children and their families (Brugnaro et al. 2024; AlShatti et al. 2021; Williams et al. 2018; Cabrera et al. 2022).

Considering that developmental transitions influence both the child's functional abilities and the family's experiences, it is anticipated that caregivers will have different perceptions when raising children compared to those raising adolescents with DS. As adolescents seek greater independence and participation in meaningful activities, caregivers may encounter new challenges related to autonomy, education and social inclusion.

Examining caregivers' concerns and perceived impact across various developmental stages may provide valuable insights for developing age‐appropriate strategies and family‐centred interventions. Therefore, the primary objective of this study was to describe and compare the areas of concern of caregivers of children and adolescents with DS across different age ranges, as well as their impact on children's and adolescents' ability to participate in daily activities. Accordingly, this study sought to answer the following research questions:

1. What are the main areas of concern reported by caregivers of children and adolescents with DS?
2. Do caregivers' concerns and their perceived impact differ across developmental age groups?

## 2 Methods

### 2.1 Research Design

Cross‐sectional study, with nonprobability convenience sampling approved by the local ethics committee (Stigma, Caregiver, and Child with Down Syndrome: A Bioethical Analysis Protocol number 10929019.9.0000.5504), and following the recommendations of the STROBE checklist (Malta et al. 2010), and the assessments were administered according to the National Health Council Guidelines and the Declaration of Helsinki.

### 2.2 Sample Characteristics

The inclusion criteria were caregivers of children and adolescents with DS aged between 0 and 18 years, without another associated diagnosis. This age range was chosen to match the age range of the descriptive tool used. The exclusion criteria were caregivers whose child had sensory changes, such as visual or auditory not corrected by devices, that might independently affect outcomes of interest or with other known diagnoses, such as autism spectrum disorder, cerebral palsy, attention deficit and hyperactivity, in order to obtain a relatively homogeneous sample and avoid possible bias. All participants were enrolled after signing the informed consent form and obtaining the minor's assent.

### 2.3 Procedures

The online study was conducted between September and November 2022. Information was disseminated through social media at the Federal University of São Carlos and other private and public healthcare institutions that provide therapeutic services for children and adolescents with DS. A demographic questionnaire and the 19‐item About My Child (AMC) questionnaire were administered via electronic form by a single evaluator. In case caregivers had some doubts, these were resolved remotely through Messenger text or audio via WhatsApp.

### 2.4 Measurement Instrument

#### 2.4.1 Demographic Questionnaire

A demographic questionnaire was used to collect information about the child's sex, age, caregiver–child relationship (e.g., mother, father and sibling) and caregiver age, with the purpose of describing the sample's characteristics.

#### 2.4.2 AMC

AMC is a tool based on the World Health Organization's International Classification of Functioning, Disability and Health (ICF) that aims to assess the family's perception of strengths and concerns about participation in daily activities. The questionnaire is self‐completed by caregivers of children and adolescents aged 0 to 18 years, with an average completion time of 10 min. Its psychometric properties are well‐established (Williams et al. 2018; Ritzema et al. 2016).

This study used the 19‐item version of AMC (Ritzema et al. 2016). The questions focus on the child's functioning. The caregiver indicates whether they are *concerned* about their child's performance in a given functional area. If the answer is ‘yes’, they are asked to report the *impact* of this concern on their child's ability to participate in everyday activities. Additional spaces are available if the parents desire to provide more information.

The AMC can be scored for two types of complexity: (i) Concern: the number of concerns (sum of the number of ‘Yes’ responses, with scores ranging from 0 to 19); a higher score represents that the caregiver has a greater number of functional concerns related to their child; and (ii) Impact: the impact of the concerns on everyday participation (the number of ‘Yes’ responses multiplied by the classification of the impact on participation [ranging from 1 = no concern to 4 = a lot] and dividing by the number of questions, resulting in the mean total impact scores ranging from 0 to 4). Greater impact represents a greater impact on children's and adolescents' daily lives due to the aspects that concern caregivers.

#### 2.4.3 Statistical Analysis

We describe the participants' characteristics (age and sex of the children/adolescents, age of the caregiver and child–caregiver relationship).

Statistical analyses were structured to address the two research questions proposed in this study. Research Question 1 (*What are the main areas of concern reported by caregivers of children and adolescents with DS?*) was answered through the median, confidence interval, mean and standard deviation of the total *Concern* and *Impact* scores and each individual item of the AMC.

Research Question 2 (*Do caregivers' concerns and their perceived impact differ across developmental age* groups?) was examined by comparing scores across the following age ranges: children: 0–2 years 11 months, 3–5 years 11 months and 6–11 years 11 months; and adolescents: ≥ 12 years. These categories were based on developmental stages described in the psychometric study of the AMC (Williams et al. 2018) and were used to explore potential age‐related differences in caregivers' concerns and perceived impacts, recognizing that family priorities and challenges evolve across developmental periods. For group comparisons, the Kruskal–Wallis test was performed due to non‐normal data distribution. Considering multiple comparisons, the Bonferroni correction was applied to the significance level (*p* < 0.05/4 = 0.013). We also compared the children and adolescent groups using the Mann–Whitney Test (significant level *p* < 0.05).

Missing values were treated using pairwise deletion, and uneven sample sizes were automatically adjusted by the SPSS software.

## 3 Results

One hundred forty primary caregivers of children and adolescents with DS were interested in participating in the study. Five were excluded for not declaring their age. Eighteen did not meet the inclusion criteria: due to the child having another health condition associated with DS (*n* = 7); because their diagnosed health condition was other than DS (*n* = 10); or DS plus the presence of sensory processing disorder (*n* = 1). In total, 117 children and adolescents with DS met the inclusion criteria and were included in the study sample. Table 1 describes the sample characteristics.

**TABLE 1:** Participant's description and caregiver's concerns in each age range.

	0–2 years 11 months	3–5 years 11 months	6–11 years 11 months	≿ 12 years
**Sex**				
Male	9	6	23	24
Female	7	3	28	17
Total	16	9	51	41
**Age**				
Mean (confidence interval)	1.44 (1.17–1.70)	4.11 (3.43–4.79)	8.45 (8.03–8.87)	14.56 (13.96–15.16)
Median	1	4	8	14
Standard deviation	0.51	0.93	1.51	1.92
**Caregivers**				
Mother	16	8	45	38
Father	0	1	2	2
Other	0	0	3	0
With no information	0	0	1	1
Mean age (SD)	39.1 (4.0)	32.0 (8.2)	44.6 (6.9)	50.7 (6.9)
**Concerns (scoring 0 to 19)**				
Mean (SD)	10.4 (5.2)	12.7 (4.2)	9.3 (5.1)	9.5 (6.4)
Item(s) with greater concerns (percentage of concerns)	1, 10, 11 (75%)	10, 19 (100%)	10 (78.4%)	11, 19 (70.7%)
Item(s) with lower concerns (percentage of concerns)	7, 8 (25%)	2 (22.2%)	2, 8 (27.5%)	6 (29.3%)
**Impact (scoring 0 to 4)**				
Mean (SD)	2.9 (0.6)	3.2 (0.4)	2.8 (0.6)	3.3 (0.7)
Item(s) with greater impact (mean of impact)	6 (3.4)	2 (4.0)	2 (3.3)	6 (3.8)
Item(s) with lower impact (mean of impact)	5 (2.0)	5 (2.9)	1 (2.5)	13 (2.9)

Abbreviation: SD, standard deviation.

### 3.1 Concerns and Impact

Throughout this section, results related to the concern and impact answers will be presented. Concerns will appear as percentages (%) of caregivers, ranging from 0% to 100%, while impact will be reported as the mean impact reported by those who reported concern about the respective item, ranging from 1 to 4.

#### 3.1.1 All Participants; *n* = 117

Considering all answers, the item with highest concern was ‘Ability to tell people what they want’, reported by 76% of the participants, while the lowest average concern was ‘Hearing’, reported by only 29% of the caregivers.

Considering average impact on participation in everyday activities, ‘Ability to sleep each night’ stands out as the highest one (3.4), reported by 32% of the caregivers. The lowest average impact was ‘Ability to dress or undress self’ (2.8), concerning 44% of the participants.

#### 3.1.2 0–2 Years and 11 Months; *n* = 16

Twelve (75%) caregivers of children aged between 0 and 2 years and 11 months indicated concerns about three items: ‘Ability to move around at home, school and community’, ‘Ability to tell people what they want’ and ‘Behavior’. Items with fewer concerns were ‘Seeing’ and ‘Hearing’ with only four (25%) caregivers reporting concerns. This could be attributed to impairments in seeing and hearing being exclusion criteria.

The items with the highest and lowest average impact on participation ratings in this age group were the same as the total group: ‘Ability to sleep each night’ (seven of the 16 caregivers) and ‘Ability to dress or undress self’ (eight of the 16 caregivers) with 3.4 and 2.0 average impact in this age group, respectively. Appendix 1 illustrates these results.

#### 3.1.3 3–5 Years and 11 Months; *n* = 9

In the 3–5 years and 11 months age group, the items ‘Ability to tell people what they want’ and ‘Participate in activities at school or in the community’ were indicated by nine (100%) caregivers as items of concern. The ‘Ability to use their hands and arms to do the things they want to do’ was highlighted as a concern by only two (22.2%) caregivers.

The item ‘Ability to use their hands and arms to do the things they want to do’ was the item with the lowest number of caregivers reporting it as a concern. The item with the lowest average impact was the item ‘Ability to dress or undress self’, in which seven of the nine (77.8%) caregivers were concerned. Appendix 2 illustrates these results.

#### 3.1.4 6–11 Years and 11 Months; *n* = 51

Caregivers of children between 6 and 11 years and 11 months indicated the item ‘Ability to tell people what they want’ as the item of greatest concern, with 40 of the 51 participants in this age range (78.4%) reporting it followed by ‘Participate in activities at school or in the community’ (76.5% of participants). The items ‘Ability to use their hands and arms to do the things they want to do’ and ‘Hearing’ caused concern in only 14 of the 51 (27.5%) caregivers of children in this age group.

For these caregivers, the highest average impact was for the item with the lowest concern: ‘Ability to use their hands and arms to do the things they want to do’, with an average of 3.3. The item with the lowest average impact (2.5) was ‘Ability to move around at home, school and community’, without presenting a high level of concern (41.2% of caregivers). Appendix 3 illustrates these results.

#### 3.1.5 ≿ 12 Years; *n* = 41

For caregivers of adolescents ≿ 12 years, the concerns ‘Behavior’ and ‘Participate in activities at school or in the community’ stand out, reported by 29 of the 41 (70.7%) caregivers. At the other end of the concern spectrum, the item ‘Ability to sleep each night’ was highlighted by only 12 of the 41 (29.3%) caregivers.

The highest average impact rating for caregivers of adolescents ≿ 12 years was for the item with the least concern: ‘Ability to sleep each night’, with an average of 3.8. The lowest average impact (2.9) was for item ‘Pain’, which presented 39.0% of concern among caregivers. Appendix 4 illustrates these descriptive results.

Analyses were conducted to check if there was a pattern of concern in more than one item for the same caregiver. It was found that when caregivers reported sleep concerns, 95% (36 of 38 caregivers) also reported behaviour concerns, ranging from 86% (six of seven caregivers of children aged 0–2 years and 11 months) to 100% (12 of 12 caregivers of adolescents age ≿ 12 years). In addition, 48 of 57 caregivers (84%) who reported concerns about ‘Ability to move around at home, school, and community’ also reported concern about ‘Participate in activities at school or in the community’.

Among caregivers who reported communication concerns (89 of 117—total caregivers), 62% of them reported concerns with participating at home and 85% of them reported concerns about participating at school or in the community. Table 2 presents the items with concurrent concerns.

**TABLE 2:** Simultaneous concerns between two items.

	0–2 years 11 months	6–11 years 11 months	≿ 12 years	Total
**Sleep and behaviour**				
Sleep concern	7	15	12	38
Behaviour concern among caregivers who had reported sleep concern	6	14	12	36
Percentage	86%	93%	100%	95%
**Mobility and participation at school or in the community**				
Mobility concern	12	21	21	57
Participation at school or in the community among caregivers who had reported mobility concern	8	19	18	48
Percentage	67%	90%	86%	84%
**Communication and participation at school or in the community**				
Communication concern	12	40	28	89
Participation at school or in the community among caregivers who had reported communication concern	8	35	24	76
Percentage	67%	87%	86%	85%
**Communication and participation at home**				
Communication concern	12	40	28	89
Participation at home among caregivers who had reported communication concern	8	20	19	55
Percentage	67%	50%	68%	62%

*Source:* Developed by the authors.

*Note:* The age range between 3 and 5 years and 11 months was not considered individually due to the low number of participants but was included in the total analysis.

### 3.2 Comparison of Concerns and Impact Across the Age Range

The statistical analysis showed that there were no statistically significant differences across age ranges for either the concerns (*p* = 0.313) or impact (*p* = 0.256), nor for the comparison between children and adolescent groups (concerns: *p* = 0.348; impact: *p* = 0.174). Table 3 illustrates the statistical results.

**TABLE 3:** Comparison of concern and perceived impact across different age ranges.

Variables	0–2 years 11 months	3–5 years 11 months	6–11 years 11 months	≥ 12 years	H statistic	*p*
	Median (CI)	Median (CI)	Median (CI)	Median (CI)	Kruskal–Wallis test	
**Concern**	11 (7.62–13.13)	12 (9.43–15.91)	9 (7.85–10.7)	9 (7.43–11.49)	3.559	0.313
**Impact**	29 (21.28–37.97)	34 (27.42–54.8)	25 (22.35–32.23)	28 (24.24–38.88)	4.047	0.256
	**Children** **Median (CI)**	**Adolescents** **Median (CI)**	**—**	**—**	**Mann–Whitney *U***	***p*‐value**
**Concern**	9.5 (8.75–11.06)	9 (7.43–11.49)	**—**	**—**	55.550	0.348
**Impact**	26 (25.41–33.43)	28 (24.24–38.88)	**—**	**—**	48.000	0.174

Abbreviation: CI = confidence interval.

Although no statistically significant differences were observed, we performed a descriptive analysis to illustrate which items presented the highest and lowest levels of concern and impact. Appendix 5 provides a representation of these results.

## 4 Discussion

The primary objective of this study was to describe and compare the areas of concern of caregivers of children and adolescents with DS across different age ranges, as well as their impact on children's and adolescents' ability to participate in daily activities. The items that caregivers were most concerned about were the ‘Ability to tell people what they want’, ‘Participate in activities at school or in the community’ and ‘Behavior’. Similar results were reported in the study of children with development needs between 0 and 5 years old by Pratte et al. (2020), which reported that most caregivers were concerned about the communication, affective and behavioural skills of their children. They also showed that mothers, parents with lower incomes and older parents had a significantly higher percentage of concerns about communication skills than other parents. These findings are not surprising, as children and adolescents with DS frequently face challenges in their language development, which can lead to noticeable delays in their communication skills. These delays are particularly evident in specific areas, such as correctly assembling words and formulating coherent sentences. As a result, they may struggle with expressing their thoughts and ideas effectively, impacting their overall social interactions and academic performance and perhaps leading to behavioural issues related to frustration. Understanding these challenges is essential for providing the appropriate support and resources to help these children enhance their language abilities (Grieco et al. 2015). At the same time, behavioural issues also occur as a characteristic of persons with DS. Studies indicate that children and adolescents with DS tend to have more social interactions in their early years compared to typically developing peers (Brugnaro et al. 2022; Brugnaro et al. 2024). However, as they grow older and the need for behavioural regulation increases, some behavioural challenges may arise, including stubbornness, argumentativeness and decreased attention to tasks (Grieco et al. 2015).

Many studies have reported that children and adolescents with disabilities experience difficulties with their sleep patterns. Parents are concerned about this, especially because their children cannot perform well during the day and at school. This can be explained by the fact that children and adolescents with DS experience difficulty in sleep initiation and sleep‐disordered breathing that affects daytime functioning (Horne et al. 2019). This also affects caregivers, who change their routines to adapt to their child (AlShatti et al. 2021; Freitas et al. 2017; Cabrera et al. 2022). The item that appeared most frequently for the 0–2 years and 11 months age group was ‘Ability to move around at home, school, and community’, as it was reported by 75% of the caregivers of children in this youngest age range. This may be because children with DS between 3 and 5 years and 11 months can walk and are improving mobility, such as climbing stairs, reducing the concern among caregivers, despite the delayed gait development in children with DS (Jain et al. 2022; Winders et al. 2018). Participation in activities can raise concerns, as children and adolescents with disabilities often have lower participation rates compared to typically developing peers. Additionally, they frequently require social environmental support from others during these activities (King et al. 2013). Parents may observe these challenges in their child's daily activities, which can lead to significant concern for caregivers (MacDonald et al. 2016).

Looking at the lowest percentages, there is more heterogeneity in the distribution of items among age groups. The items ‘Hearing’, ‘Ability to sleep each night’, ‘Ability to use their hands and arms to do the things they want to do’ and ‘Seeing’ appeared as the least frequent concerns of all age groups. Despite fewer reported concerns for these items, most of them showed greater mean impacts for caregivers in all age groups. This may be due to the fact that these items represent common characteristics of children and adolescents with DS, and caregivers already expect children to have these as a part of their functioning. This matching between expectation and reality causes them less *concern* about their children's condition, but not less *impact*, as these impairments may generate more challenging situations along the children's development.

Another important finding is that the distribution of the impacts is not consistent among age or items. Similar to the concerns, they vary by age and developmental stages. For example, ‘Ability to move around at home, school and community’ shows a 3.7 impact rating for caregivers of children (3–5 years and 11 months), but only 2.5 for the next age range. Parents may be concerned about their children's mobility due to the challenging situations they face during their participation in the school and community settings, and they are less concerned about activities in which they could support their children (Jain et al. 2022; Camden et al. 2020).

Another important finding is that, although no statistically significant differences were observed across developmental age groups, the distribution of perceived impact was not consistent across ages or items. Similar to caregiver concerns, the perceived impact appears to vary according to specific functional domains and developmental contexts rather than age alone. For instance, the item ‘Ability to move around at home, school, and community’ showed a higher perceived impact among caregivers of younger children (3–5 years and 11 months; mean impact = 3.7) compared with the subsequent age group (mean impact = 2.5). This pattern suggests that caregivers may perceive mobility‐related challenges as more impactful during early childhood, when children are increasingly exposed to school and community participation demands that require environmental navigation and autonomy. As children grow older, caregivers may report lower perceived impact in domains where they can provide compensatory support or where routines become more established. These findings align with previous literature indicating that caregiver perceptions are shaped by participation demands and environmental expectations rather than by developmental age (Jain et al. 2022; Camden et al. 2020). From a functioning‐oriented perspective, this reinforces the importance of examining item‐level patterns to better understand how specific activities and participation contexts influence caregiver experiences across developmental stages (World Health Organization 2001; Rosenbaum and Gorter 2012).

### 4.1 Clinical Implications and Conclusion

Considering the heterogeneity of the distribution of concerns and impacts, the care of children and adolescents with DS should match specific needs that may change over the lifespan and vary among individuals. The value of a measure like AMC is that it offers parents and service providers a structured approach to address caregiver perspectives about daily life and how much impact impairments or limitations might have on their participation.

Health professionals should be encouraged to look at each child and family individually. The data obtained from this study may help to inform clinical practice decisions when collaborating with children and adolescents with DS and their families.

Additionally, the AMC benefits caregivers, allowing them to recognize their primary concerns and seek support from appropriate professionals. This leads to more effective guidance and intervention for the child's care and also provides a basis for facilities to develop appropriate service delivery resources.

In conclusion, the AMC enables professionals to tailor their approaches, provide family‐centred care and promote family participation in the treatment process. With a clearer understanding of their concerns, families can set more focused goals and actively engage in their child's care.

### 4.2 Strengths and Limitations

This study explored the concerns and daily life impacts reported by caregivers of children and adolescents with DS using the AMC tool, which considers biopsychosocial aspects of children and adolescents' lives and their families. This approach allows caregivers, therapists and researchers to explore important concerns and their impact on daily life. We interpret the differentiation of the impacts reported along the different age groups as a good proof‐of‐concept of the utility of the AMC in clinical practice.

There are some limitations of this study. First, the small sample size of caregivers of children aged between 0 and 6 years old, which limits our understanding of this young group. Second, the nonprobability convenience sample recruited via social media and healthcare institutions could make interpretation and generalization of the findings difficult. Third, we do not collect longitudinal data enabling the comparison across age groups. Last, an additional qualitative data collection with triangulation of caregiver‐reported data from other sources could provide a better interpretation of the findings. Since the collection of the data, a youth version of the AMC called ‘About Me’ has been published, and it would have been interesting to collect the self‐reported concerns from the adolescents (https://canchild.ca/shop/about‐me/). This might be done in future studies.

## Author Contributions

**Beatriz Helena Brugnaro:** conceptualization, investigation, funding acquisition, writing – original draft, writing – review and editing, visualization, methodology, validation, formal analysis, data curation, resources. **Henrique Granado Jábali:** writing – original draft, writing – review and editing, methodology, data curation, formal analysis. **Rosa Isabel Fonseca Angulo:** writing – original draft, writing – review and editing, data curation. **Rafaela Campos:** writing – original draft, methodology, data curation. **Marilyn Wright:** writing – original draft, methodology, validation, writing – review and editing, data curation. **Rachel Teplicky:** writing – original draft, writing – review and editing, methodology, validation, data curation. **Peter Rosenbaum:** writing – original draft, methodology, writing – review and editing, visualization, data curation. **Olaf Kraus de Camargo:** writing – original draft, validation, methodology, writing – review and editing, data curation, supervision. **Nelci Adriana Cicuto Ferreira Rocha:** data curation, supervision, resources, project administration, visualization, writing – review and editing, methodology, validation, investigation, writing – original draft, funding acquisition, conceptualization.

## Funding

This work was supported by São Paulo Research Foundation (FAPESP) [grants 2019/13716‐0, 2019/13570‐6 and 2021/15016‐6] and Coordination for the Improvement of Higher Education Personnel (CAPES) [financial code 001].

## Ethics Statement

The local Ethics Committee approved this work for Human Research (CAAE: 10929019.9.0000.5504).

## Conflicts of Interest

The authors declare no conflicts of interest.

## Data Availability

The data that support the findings of this study are available from the corresponding author upon reasonable request.

## Appendix 1 Concerns Percentage and Impact Per Item—Answers From Caregivers of Children From 0 to 2 Years and 11 Months

*Source:* Developed by the authors.

## Appendix 2 Concerns Percentage and Impact Per Item—Answers From Caregivers of Children From 3 to 5 Years and 11 Months

*Source:* Developed by the authors.

## Appendix 3 Concerns Percentage and Impact Per Item—Answers From Caregivers of Children From 6 to 11 Years and 11 Months

*Source:* Developed by the authors.

## Appendix 4 Concerns Percentage and Impact Per Item—Answers From Caregivers of Children of ≿ 12 Years

*Source:* Developed by the authors.

## Appendix 5 Percentage of Caregivers Concerned Per Item for Total of the Participants, in Each Group (Children and Adolescents) and Percentage Between the Groups

**Table:**

Item	Caregivers concerned (total)	Children's caregivers concerned	Adolescents' caregivers concerned	Impact (total)	Children's caregivers mean impact	Adolescents' caregivers mean impact
	%	%	%	Mean (median)	Mean (median)	Mean (median)
Locomotion	48.72	47.37	51.22	2.96 (3)	2.72 (3)	3.38 (4)
Use of hands and arms	33.33	32.89	34.15	3.25 (4)	3.27 (4)	3.21 (3)
Feeding/eating	43.59	46.05	39.02	2.96 (3)	2.77 (3)	3.38 (4)
Toileting	52.99	55.26	48.78	2.87 (3)	2.66 (3)	3.30 (4)
Dressing	43.59	50.00	31.71	2.78 (3)	2.58 (3)	3.38 (4)
Sleep	32.48	34.21	29.27	3.38 (4)	3.22 (3)	3.75 (4)
Seeing	37.61	36.84	39.02	3.07 (3)	2.86 (3)	3.44 (4)
Hearing	29.06	27.63	31.71	3.12 (4)	3.00 (3)	3.31 (4)
Comprehension	53.85	57.89	46.34	3.08 (3)	2.89 (3)	3.53 (4)
Communication	76.07	80.26	68.29	3.24 (3)	3.18 (3)	3.36 (4)
Behaviour	73.50	75.00	70.73	3.05 (3)	2.95 (3)	3.24 (4)
Mood	46.15	43.42	51.22	3.11 (3)	2.94 (3)	3.38 (4)
Pain	44.44	47.37	39.02	2.90 (3)	2.89 (3)	2.94 (3)
Learning	65.81	67.11	63.41	3.25 (3)	3.16 (3)	3.42 (4)
Memory	63.25	64.47	60.98	3.07 (3)	2.98 (3)	3.24 (3)
Relationship with children	58.97	57.89	60.98	3.29 (4)	3.16 (3)	3.52 (4)
Relationship with adults	44.44	39.47	53.66	3.06 (3)	2.93 (3)	3.23 (4)
Home participation	52.14	50.00	56.10	3.00 (3)	2.92 (3)	3.13 (3)
School and community participation	75.21	77.63	70.73	3.23 (3)	3.17 (3)	3.34 (4)

*Note:* Difference between groups is the absolute difference obtained by the subtraction of the percentage of children's caregivers concerned by the percentage of adolescents' caregivers concerned for each item. Negative values indicate greater concern for adolescents' caregivers.
